# A ParDE-family toxin antitoxin system in major resistance plasmids of Enterobacteriaceae confers antibiotic and heat tolerance

**DOI:** 10.1038/s41598-019-46318-1

**Published:** 2019-07-08

**Authors:** Muhammad Kamruzzaman, Jonathan Iredell

**Affiliations:** 1 Centre for Infectious Diseases and Microbiology, The Westmead Institute for Medical Research, The University of Sydney, Westmead, New South Wales Australia; 2 Westmead Hospital, Westmead, New South Wales Australia

**Keywords:** Bacteriology, Bacterial infection

## Abstract

Toxin-antitoxin (TA) systems were initially discovered as plasmid addiction systems on low-copy-number plasmids. Thousands of TA loci have since been identified on chromosomes, plasmids and mobile elements in bacteria and archaea with diverse roles in bacterial physiology and in maintenance of genetic elements. Here, we identified and characterised a plasmid mediated type II TA system in Enterobacteriaceae as a member of the ParDE super family. This system (hereafter, ParDE^I^) is distributed among IncI and IncF-type antibiotic resistance and virulence plasmids found in avian and human-source *Escherichia coli* and *Salmonella*. It is found that ParDE^I^ is a plasmid stability and stress response module that increases tolerance of aminoglycoside, quinolone and β-lactam antibiotics in *E. coli* by ~100–1,000-fold, and thus to levels beyond those achievable in the course of antibiotic therapy for human infections. ParDE^I^ also confers a clear survival advantage at 42 °C and expression of the ParE^I^ toxin *in trans* induces the SOS response, inhibits cell division and promotes biofilm formation. This transmissible high-level antibiotic tolerance is likely to be an important factor in the success of the IncI and IncF plasmids which carry it and the important pathogens in which these are resident.

## Introduction

Self-transferable (conjugative) antibiotic resistance plasmids promote their own retention in bacterial populations via “addiction systems”, typically toxin-antitoxin (TA) modules that inhibit or kill plasmid-free daughter cells and serve to maintain plasmids in bacterial populations. The antibiotic resistance conferred on major pathogens such as *Escherichia coli* and *Klebsiella pneumoniae* in this way is a major problem worldwide^1,2^.

The archetypal TA system is a two-gene operon, producing a protein toxin and an antitoxin that opposes it in the bacterial cell^3^. TA systems were first recognised by their role in plasmid maintenance by post segregational killing of plasmid-free cells^4^. In a similar way, TA systems found on other mobile genetic elements like transposons, prophages and genomic islands also contribute to their maintenance^5–7^. Hundreds of TA systems have been identified in bacterial and archaeal chromosomes but the functions of many remain elusive. It is reported that numerous chromosomal TA systems contribute to bacterial stress responses, biofilm formation, intestinal colonisation, virulence, antibiotic tolerance and persister cell formation^8–12^. The roles of chromosomal TA systems in antibiotic tolerance and persister cell formation have received a great deal of attention^8,9,13,14^ although the molecular mechanism behind persister formation is not fully understood^14^.

Persister cells are a phenotypic subpopulation of bacteria that form under different environmental cues and remain viable after exposure to usually lethal concentrations of antibiotics^15^. Antibiotic tolerance is important in the survival of bacterial biofilms. Several chromosomal TA systems are induced in biofilms^11^, where persister cells are implicated in antimicrobial tolerance and treatment failure^16,17^.

TA system toxins typically exert their function by impairing bacterial replication or RNA translation^3,18,19^ and are commonly divided into several types, the most common and best understood of which are the type II TA systems, with protein toxin-antitoxin pairs. In classic type II TA systems, the antitoxin is encoded upstream of the toxin and the antitoxin protein usually forms a dimer, each monomer generally being composed of an N-terminal dimerisation and DNA-binding domain and a C-terminal domain for toxin binding and neutralisation^20^ (Fig. S1). Chromosomal type II TA systems are thought to be heavily involved in antibiotic tolerance and formation of persister cells^8,9,15^ but there is ongoing debate arising from published data^13,14,21–25^. Recent studies^24,25^ have shown no effect on antibiotic tolerance or persister cell formation from the deletion of as many as ten *E. coli* chromosomal type II TA systems, but other *E. coli* type II TA modules such as *vapBC* and *pasTI* have been shown to increase persister cell formation upon exposure to different antibiotics^26,27^ and mutations in some TA modules also enhance the formation of antibiotic-tolerant persisters^28,29^. Expression of (type II) chromosomal RelE, MazF and *E. coli* F plasmid-borne CcdB toxins in the absence of their cognate antitoxins (RelB, MazE and CcdA respectively) have been shown to increase antibiotic survival^30–32^ and other types of TA systems including TisB-istR-1 and GhoST have also been shown to be involved in persistence in *E. coli*^33,34^ but their exact molecular mechanisms are largely unknown.

One of the best-known systems is the chromosomal Type II TA system RelBE, homologues of which are widely distributed in Gram-positive and Gram-negative bacterial chromosomes and in the archaea, where they appear to be important in environmental and nutrient stress adaptation^35^. ParDE, another type II TA system, was first identified in the broad-host range plasmid RK2 as a plasmid maintenance/stability (“addiction”) system^36^. The ParE toxins are in the RelE toxin superfamily and have significant primary DNA sequence homologies with RelE but distinct cellular targets and mechanisms of action. ParE is a gyrase inhibitor that blocks DNA replication^37^ while RelE is an endoribonuclease that inhibits translation by promoting cleavage of mRNA in the ribosomal A-site^38–40^. Here we describe a RelE/ParE superfamily toxin-containing TA system in IncI and IncF conjugative plasmids, these plasmids being major vectors of β-lactam antibiotic resistance in the Enterobacteriaceae. Our results indicate that this ParDE^I^ TA system is a plasmid maintenance system with an additional role in bacterial stress management particularly in antibiotic tolerance and heat tolerance. This TA system is therefore expected to contribute to the success of IncI and IncF plasmids in the global spread of antibiotic resistance and may play a vital role in treatment failure of major classes of antibiotics.

## Results

### A ParDE-type TA system in IncI1 plasmid pJIE512b

A putative type II TA system with a RelE-like toxin was predicted in our sequenced IncI1 plasmid pJIE512b (Accession No. HG970648.1) by TA-finder (http://202.120.12.133/TAfinder/index.php). A BLASTp search revealed identical toxin proteins in GenBank, variably annotated as ‘putative hypothetical protein’, YacB, RelE toxin, RelE/ParE family protein, RelE/StbE family protein, plasmid stabilisation system protein, mRNA interferase toxin RelE or RelE/StbE. Although the amino acid sequence is only 18%, 15% and 15% identical with *E. coli* chromosomal RelE, plasmid RK2 ParE and *Caulobacter crescentus* chromosomal ParE1 (ParE1-CC, hereafter), respectively (Fig. S2a), the predicted secondary structure of the putative toxin (α1α2β1β2β3) is closer to the secondary structure of ParE1-CC (α1α2β1β2) than RK2 ParE (α1β1β2α2) and RelE (β1α1α2α3β2β3β4α4) (Fig. S2b). Conserved domain search for the toxin protein identifies it as a member of ParE toxin superfamily, and three highly conserved amino acids in RelE (61R, 81R and 87Y) that are thought to be important for mRNA cleavage^39^ are conspicuously absent from the putative toxin and from other ParE toxins (Fig. S2a).

The putative antitoxin protein is predicted as RHH-like by TA-finder. BLASTp search of GenBank found a ‘hypothetical protein’, ATPase component of ABC transporter, ‘prevent host death’ protein, addiction module antitoxin RelB/DinJ family protein, RHH domain protein, Phd antitoxin, TA system protein, etc. The amino acid sequence has 15%, 11% and 21% identity with *E. coli* chromosomal RelB, plasmid RK2 ParD and *Caulobacter crescentus* chromosomal ParD1 (ParD1-CC, hereafter), respectively (Fig. S3a), and the predicted secondary structure (β1α1α2α3) is the same as ParD1-CC^41^, but different from *E. coli* RelB (β1α1α2α3β2β3β4α4) and RK2-ParD which has an additional α-helix (β1α1α2α3α4) at the C-terminus (Fig. S3b). A conserved domain database search identifies the putative antitoxin as a COG3905 superfamily protein, although it has the ribbon-helix-helix (RHH) motif of the ParD and RelB antitoxins. Amino acid sequence and predicted secondary structure places the putative antitoxin closest to the ParD antitoxin and we will therefore use the term ParDE^I^ (the superscript^I^ denoting initial recognition in an IncI plasmid).

### Distribution of ParDE^I^

**Figure 1:**
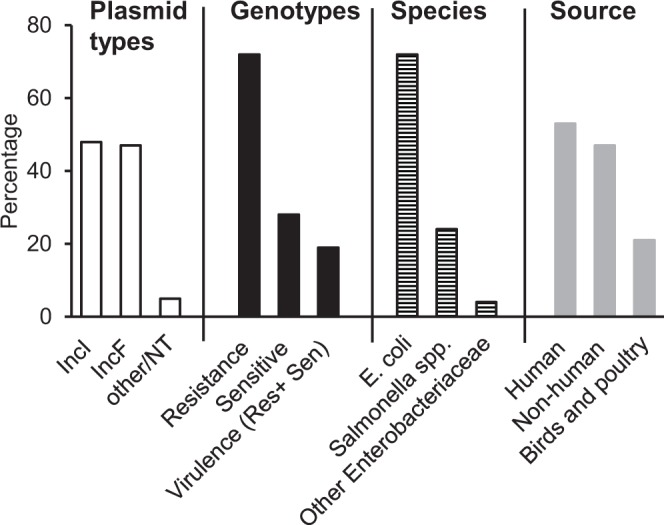
Distribution of the ParDE^I^ TA system in plasmid incompatibility types (□), in antibiotic-resistance (‘resistance’), plasmids without identified antibiotic resistance genes (‘sensitive’) and plasmids identified as “virulence plasmids” (■); and the bacterial hosts of those plasmids (**⊟**) and their sources ().

A BLASTn search using the complete coding region for ParDE^I^ identified 153 plasmid sequences (as on October 2018) in GenBank that included at least 80% nucleotide sequence identity and 90% of the length of ParDE^I^ coding region. Searching for *rep* genes and information available in published sequences and papers indicated that 48% (74) and 47% (72) of plasmids were of IncI and IncF type, respectively, and most from *E. coli* (72%) and *Salmonella species* (24%) (Fig. 1 and Table S1). The sources of those isolates were identified as birds (especially poultry) and animals (39%), vegetables and foods (5%) and human samples (53%). 72% of plasmids carrying ParDE^I^ have at least one and many have multiple antibiotic resistance genes. 19% of plasmids in which the ParDE^I^ system was identified (irrespective of antibiotic resistance) carried important virulence genes including shiga toxin and colicins (Fig. 1 and Table S1).

### ParDE^I^ TA is a plasmid maintenance system

**Figure 2:**
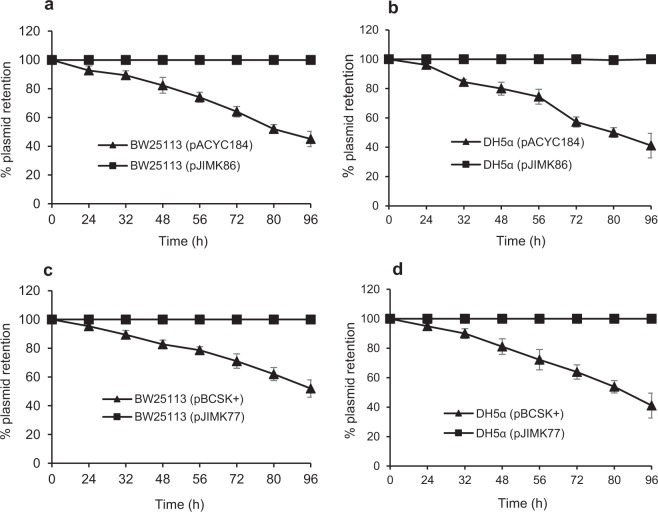
Plasmid stability assay. Plasmid stability was assessed for cloned ParDE^I^ TA system in low copy (**a**,**b**) and high copy number plasmids (**c**,**d**) in *E. coli* BW25113 (**a**,**c**) and DH5α (**b**,**d**), expressed as relative plasmid retention over time. Plasmids carrying cloned ParDE^I^ TA were very stable (■), irrespective of plasmid type and host strain, while ~60% of control plasmids (vector only) were lost (▲) within 96 h without antibiotic selection. Each  experiment was repeated three times, and the mean values with standard deviations (error bars) are presented.

The BPROM^42^ software was used to predict the −35, −10 promoter sequences and the ribosome binding site (RBS) of *parDE*^*I*^ (Fig. S4). *parDE*^*I*^ was cloned with its putative promoter and RBS into low (pACYC184) and high copy (pBCSK^+^) vectors and plasmid stability assayed in two *E. coli* K12 strains, DH5α (*recA* mutant) and BW25113 (*recA* proficient wild type). ParDE^I^ ensured complete retention of both plasmids in antibiotic free media in both strains, while control plasmids lacking ParDE^I^ were lost from 50–60% of cells within 96 h (Fig. 2). This indicates that ParDE^I^ is an effective plasmid maintenance (addiction) system.

### Effects of the ParE^I^ putative toxin on bacterial growth and cell morphology

**Figure 3:**
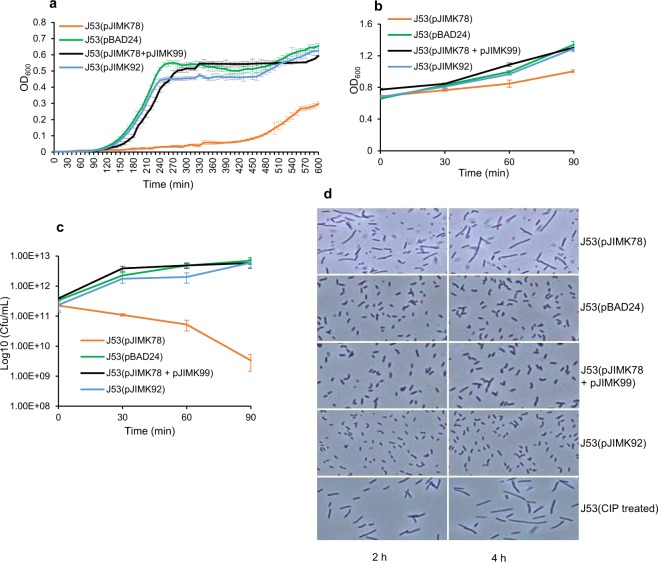
Effects of ParE^I^ toxin on *E. coli* growth, survival and cell morphology. J53(pJIMK78) growth is significantly inhibited compared to J53(pBAD24) empty vector, J53(pJIMK92) expressing the truncated ParE^I^ toxin and J53(pJIMK78 + pJIMK99) expressing both toxin and antitoxin, respectively, with ParE^I^ continually expressed from beginning of culture (**a**). Optical density (**b**) and viable bacterial count (**c**) were also reduced after toxin induction in mid-logarithmic phase. (**d**) Elongated cell morphology in J53(pJIMK78) with ParE^I^ toxin expressed (top panel) is similar to CIP-treated (0.25 µg/mL) J53 cells (bottom panel), while normal cell morphology is observed in the presence of the truncated toxin (2nd panel from bottom) or empty vector (2nd panel from top) or with the co-expression of toxin and antitoxin (3rd panel from top). Each experiment was repeated three times, and the mean values with standard deviations (error bars) are presented.

To examine the effects of ParE^I^ toxin on bacterial growth, *parE*^*I*^ and *parD*^*I*^ coding regions were cloned under the control of the arabinose-inducible promoter of pBAD24 and pBAD33-Gm vectors to construct pJIMK78 and pJIMK99 respectively. A C-terminal truncated version of *parE*^*I*^ toxin gene was cloned into pBAD24 under the control of the arabinose-inducible promoter to construct pJIMK92. Plasmids pBAD24^43^ (with pBR322 *ori* and an ampicillin resistance marker) and pBAD33-Gm^44^ (p15A *ori* and a gentamicin resistance marker) are expression vectors with arabinose-inducible promoters but different replication systems and resistance markers and thus allow controlled simultaneous expression of both toxin and antitoxin gene in the same bacteria. Induction of *parE*^*I*^ expression from J53(pJIMK78) extended lag phase and inhibited growth (Fig. 3a). The effect of ParE^I^ toxin on growth was neutralised when both toxin and antitoxin were simultaneously expressed *in trans* from J53(pJIMK78 + pJIMK99) after induction with arabinose. A truncated or non-toxic version of *parE*^*I*^ was generated by removing amino acid residues from the C-terminus region of ParE^I^ known to be essential for stability and toxicity of ParE protein^45^. Expression of truncated ParE^I^ toxin from J53(pJIMK92) with 0.2% arabinose did not result in growth inhibition. OD_600_ and viable cell counts were both decreased after *parE*^*I*^ was induced in exponentially growing J53 cells (Fig. 3b,c) compared to simultaneously expressed toxin and antitoxin and compared to expression of truncated toxin. Induction of ParE^I^ toxin produced elongated *E. coli* cell morphology (Fig. 3d) which is very similar to that of ciprofloxacin-treated J53 cells and to that ascribed to DNA gyrase inhibition by other ParE toxins^45,46^ but normal cell morphology was observed from cells in which both toxin and antitoxin genes were co-expressed *in trans* or those in which only truncated toxin was expressed (Fig. 3d).

### ParE^I^ triggers the SOS response

**Figure 4:**
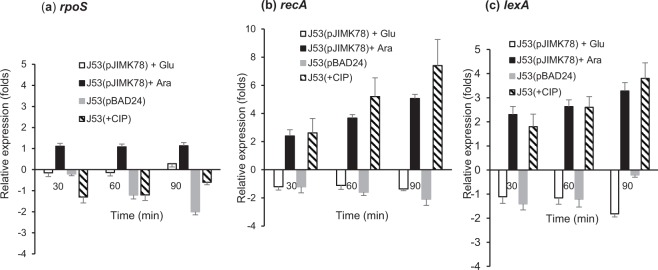
Relative expression of stress response genes *rpoS* (**a**), *recA* (**b**) and *lexA* (**c**), in the presence of ParE^I^ toxin induction or repression in *E. coli* J53(pJIMK78). Toxin-induced state (with 0.2% arabinose) (■), toxin-repressed state (with 0.2% glucose) (□) is compared with CIP-treated (0.25 µg/mL) cells () and J53(pBAD24) vector-only control cells (). Each experiment was repeated three times, and the mean values with standard deviations (error bars) are presented.

Unopposed ParE expression in *V. cholerae* is associated with elongated cell morphology, DNA damage and induction of the SOS response^46^. In addition to cell elongation, *parE*^*I*^ expression from pJIMK78 *in trans* resulted in significantly increased *recA* and *lexA* gene expression in *E. coli*, consistent with induction of the SOS response (Fig. 4b,c). We found no significant increase in *rpoS* mRNA (Fig. 4a) to indicate general stress response (GSR) pathway induction.

### ParDE^I^ provides antibiotic tolerance and promotes persister cell formation

**Figure 5:**
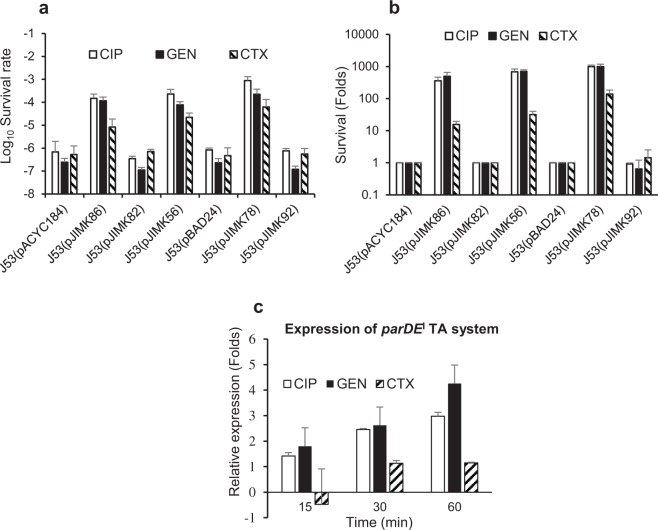
Role of ParDE^I^ TA system in antibiotic tolerance/persister cell formation. Survival after 5 h exposure to high doses of antibiotics CIP (□), GEN (■) and CTX (). *E. coli* J53 cells carrying ParDE^I^ (cloned in pJIMK86 or from the native plasmid pJIMK56) or ParE^I^ toxin alone (pJIMK78) induced with 0.2% arabinose increased survival by ~100 to 1,000-fold for CIP and GEN and ~10 to 100-fold for CTX; expressed as (**a**) relative survival and (**b**) as fold-change. (**c**) Expression of ParDE^I^ TA genes after exposure to sub-MIC concentration of different antibiotics (CIP, GEN and CTX antibiotic) is increased 3–4 folds within 60 min of CIP and GEN exposure but only slightly after CTX exposure. Antibiotic tolerance experiments (**a**,**b**) were repeated five times and gene expression experiment (**c**) was repeated 3 times. Mean values and standard deviations (error bars) are shown.

Bacterial tolerance of ciprofloxacin (CIP, a fluoroquinolone), gentamicin (GEN, an aminocyclitol aminoglycoside) and cefotaxime (CTX, a third-generation cephalosporin) were assessed in *E. coli* J53 with or without *parDE*^*I*^ and after specific activation of full-length or truncated ParE^I^ toxin. Experiments were performed five times independently in supplemented M9 media (described in the methods section) and tolerance was assessed at 3 h and 5 h after exposure to high doses of antibiotics. The baseline MIC of *E. coli* J53 was low (very susceptible) to all 3 antibiotics tested (MIC = 0.25 µg/mL, 0.25 µg/mL and 0.064 µg/mL for CTX, GEN and CIP antibiotics, respectively). There was no change in MIC between J53 alone and with recombinant constructs used in antibiotic tolerance assay for above mentioned antibiotics. We compared (i) pJIMK86 (with cloned *parDE*^*I*^) with pACYC184 (low-copy vector-only control), (ii) a naturally-occurring antibiotic-susceptible IncI1 plasmid with (pJIMK56) or without (specific deletion of) *parDE*^*I*^ (pJIMK82) and (iii) pJIMK78 (*parE*^*I*^ under control of an arabinose-inducible promotor), pJIMK92 (C-terminus truncated *parE*^*I*^ toxin, under control of arabinose-induced promoter) and with pBAD24 (vector-only control), all in a J53 *E. coli* background. All bacteria expressing the ParDE^I^ TA system had a clear survival advantage in the presence of high concentrations of all 3 antibiotics at both time points examined (Figs 5a, S5a), with at least ~1,000-fold better survival of supra-MIC CIP (5 µg/mL) and GEN (16 µg/mL) exposure and at least ~10-fold better survival of supra-MIC CTX (16 µg/mL) exposure (Figs 5b, S5b). Specific activation of toxin alone (without the putative antitoxin ParD^I^) provided the same or slightly superior survival advantage than ParDE^I^ together (~1,000 fold) in the presence of high concentrations of CIP and GEN and at least ~100-fold better survival in the presence of high concentration of CTX (Figs 5a,b, S5a,b). Expression of truncated toxin from pJIMK92 did not show any additional survival advantage in *E. coli* than are those carrying empty vector (Figs 5a,b, S5a,b). Note that these experiments were performed with pJIMK56 and pJIMK82, these being derivatives of pJIE512b from which we have deleted the powerful antibiotic resistance gene *bla*_CMY-2_^47,48^.

Expression of *parDE*^*I*^ mRNA gradually increased with time and was 3- and 4-fold higher after 60 min exposure to *sub*-MIC concentrations of CIP (0.05 µg/mL) and GEN (0.20 µg/mL), respectively (Fig. 5c). Expression of *parDE*^*I*^ was initially downregulated at 15 min and only slightly increased after 30 or 60 min exposure to *sub*-MIC concentrations of CTX (0.20 µg/mL).

### Role of ParDE^I^ in other stress conditions

**Figure 6:**
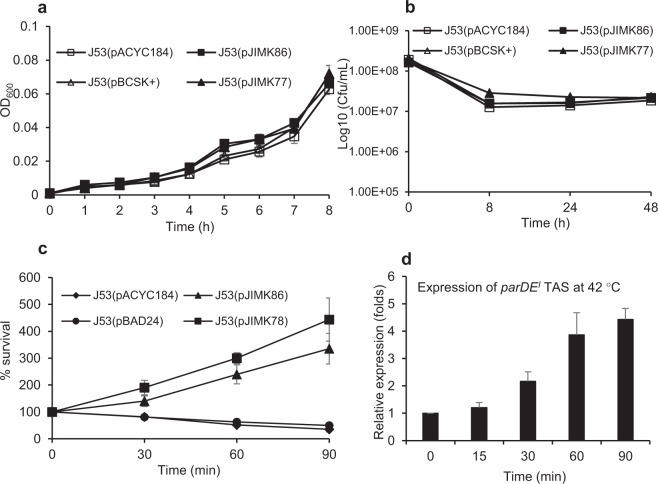
The effect of ParDE^I^ on *E. coli* (J53) growth in stress conditions. *E. coli* with cloned ParDE^I^ (pJIMK86) is not significantly different from the vector-only control in nutrient limitation (**a**) and nutrient starvation (**b**). A significant benefit is evident in *E. coli* J53(pJIMK86) and after activation of toxin from pJIMK78 (induction with 0.2% arabinose) for growth at 42 °C (**c**). Expression of ParDE^I^ is also increased at 42 °C (**d**). Each experiment was repeated 3 times and mean values and standard deviations (error bars) are presented.

The presence of ParDE^I^ appeared to make no significant difference to bacterial growth and survival in nutrient limiting (supplemented M9 containing casaminoacids but no glucose) or starvation conditions (supplemented M9, no glucose or casaminoacids) (Fig. 6a,b), but there was a significant benefit in bacterial growth was observed at 42 °C in the presence of ParE^I^ toxin alone and ParDE^I^ together. In the absence of ParDE^I^ or ParE^I^, growth in supplemented M9 media is reduced by ~50% or more within 90 min at 42 °C (Fig. 6c). Normalised expression of *parDE*^*I*^ was also relatively increased at 42 °C, consistent with a role in the observed increased survival (Fig. 6d).

### The putative ParE^I^ toxin promotes biofilm formation in *E. coli*

Arabinose-induced ParE^I^ toxin expression in *E. coli* J53 (from pJIMK78) was associated with significantly increased biofilm formation, as determined by a simple crystal violet elution assay (Fig. S6a,b) and by direct microscopic observation (Fig. S6c). No significant biofilm formation was observed by J53 *E. coli* carrying pBAD24 vector only, or after induction of C-terminus truncated ParE^I^ toxin from plasmid pJIMK92 (Fig. S6a–c).

## Discussion

The ParDE^I^ type II TA system was mostly found in conjugative IncI and IncF plasmid types and predominantly in *E. coli* and *Salmonella species* (Fig. 1, Table S1). IncI and IncF type plasmids are major vectors of β-lactamase (AmpC, Extended-spectrum β-lactamase, Carbapenemase), aminoglycoside and quinolone resistance genes among the Enterobacteriaceae^49^. ParDE^I^ is evidently important for the stable maintenance of mobile genetic elements (here, plasmids) in *E. coli*, like other plasmid and chromosome borne ParDE-homologues^6,36^. Deletion of ParDE from chromosome II of *Vibrio cholerae*, for example, results in loss of the chromosome and death of the cell from unopposed residual toxicity of the ParE toxin^46^.

Persister cells are metabolically inactive or dormant cells which can be produced by TA toxins that inhibit vital cellular processes^34,50–52^ and are thought to be less vulnerable to (more tolerant of) antibiotics largely because of the reduced activity in the cellular processes they target^30^. Biofilm formation is often depicted in terms of a stress response^53^ and chromosomal TA systems are well known to participate in the stress response, antibiotic tolerance and persister cell formation^8,9,11,12^ but such functions have only been reported for the plasmid-borne *ccdAB* type II TA system^32^.

Studies of antibiotic tolerance and perister cell formation mediated by chromosomal type II TA systems^51^ led to some discrepancies^21,54^ and debate in recent years. Recent studies^24,25^ have confirmed no role for *E. coli* chromosomal 10 type II TA systems in antibiotic tolerance or persister cell formation and identified several factors influenced previously published persister data, including contamination of experiments by cryptic prophage, concentration of antibiotics and growth media in different studies. Taking in to account the observations from both papers^24,25^, we confirmed that our experimental strains are free from prophage ɸ80 contamination and used concentrations of antibiotics >50-fold higher than the MIC value for the CIP, GEN and CTX and performed antibiotic killing assays at two time points, 3 h and 5 h after exposure to antibiotics. The tolerance assay was performed in supplemented M9 media^25^ (in which all strains behaved consistently), but *E. coli* K12 strains (DH5α, UB5201 and J53) exhibited different and variable growth kinetics in MOPS-based media, a recommended alternative^24^.

We show here that ParDE^I^ contributes substantially to tolerance of three of the most important major classes of antibiotics for *E. coli* (Figs 5a,b, S5a,b), with >1,000-fold increased survival in the presence of supra-MIC concentrations of CIP (fluoroquinolone, targeting DNA gyrase) and GEN (aminoglycoside, targeting the 30 S ribosomal subunit) in the presence of the conjugative plasmid in which ParDE^I^ naturally occurs, and we show that this is specifically mediated by RecA-dependent SOS response by activation of the ParE^I^ toxin, similar to that found in activation of the CcdB toxin^32^. Expression of the ParE^I^ toxin alone increased bacterial survival >100-fold in the presence of CTX (cephalosporin, targeting cell wall remodelling) but while the ~10-fold increased survival in the presence of ParDE^I^ satisfies traditional definitions of ‘tolerance’ (survival at more than 4-fold MIC;^55^). ParDE^I^ expression from its natural promoter is not induced in the presence of sub-MIC CTX concentrations, in contrast to the effects of GEN and CIP (Fig. 5c). Unlike the β-lactams (e.g. CTX), the aminoglycoside and quinolone antibiotics typically result in RecA-dependent SOS stress responses, with promotion of error-prone polymerase V expression, increased biofilm formation, and increased production of metabolically inactive small cells that are relatively unaffected by antibiotics^56,57^.

ParE^I^ (the putative toxin) is therefore predicted to target DNA gyrase and participate in a RecA-dependent SOS response (Fig. 4b,c), much like the ParE1 and ParE2 toxins of *V*. *cholerae* chromosome II and the CcdB toxin of *E. coli* F-plasmids^32^. The elongated cell morphology observed after ParE^I^ expression in *E. coli* is similar to that of CIP treated cells and to that described for the ParE-toxin of ParDE-RK2 or *V. cholerae* ParE (Fig. 3d). Also consistent with a response to DNA damage, ParDE^I^ expression is induced at 42 °C (Fig. 6d), and ParDE^I^ (or the ParE^I^ toxin) markedly improves *E. coli* growth at 42 °C (Fig. 6c). ParDE^I^ does not appear to protect bacteria from nutrient stress, which ordinarily results in an RpoS-dependent general stress response (Fig. 6a,b), and we found no evidence of significantly increased *rpoS* expression in the presence of the ParE^I^ toxin (Fig. 4a). Close examination of Fig. 3a shows growth to be delayed and reduced, with an upward trend only evident 180 minutes after ParE^I^ induction in J53(pJIMK78). Similar growth trend is also seen in the same bacteria transferred to 42 °C, 180 minutes after induction of ParE^I^ toxin expression (Fig. 6c).

Detailed mechanistic studies are needed to understand the exact mechanism of ParDE^I^ mediated tolerance but this is likely to be similar to that of CcdB toxin mediated antibiotic tolerance^32^. We hypothesise that (e.g.) antibiotic stress elevates the level of Lon and/or other proteases that degrades the ParD^I^ antitoxin and releases ParE^I^ from ParD^I^-ParE^I^ toxin-antitoxin complex. The free toxin interacts with DNA gyrase to induce DNA damage and the SOS response, which in turn activates other cellular systems participating in persister cell formation.

Plasmid-borne ParDE^I^ thus increases tolerance of temperatures that might occur in febrile humans and in healthy birds. The relative frequency of *E. coli* and *Salmonella species* in poultry, foods and other non-human samples (Fig. 1) is noteworthy. *Salmonella species* are commonly associated with leafy greens, poultry meat, and eggs^58^ and are important agents of food-borne disease. Heat treatment is one of the more effective ways to eliminate *Salmonella* and other pathogens from food products^59^; pasteurisation and other heat treatments are often used in processing milk, vegetables, juices, meat, and poultry carcasses^59,60^. Plasmid-encoded temperature tolerance is thus an important acquired virulence advantage in foodborne pathogens such as *Salmonella* and *E. coli*, which must adapt to core body temperatures of ~42 °C in many avian hosts (e.g. chickens).

Finally, homologous chromosomal TA systems including RelBE, MazEF and MqsRA are known to be involved in bacterial biofilm production^12,61,62^ and the RecA-dependent SOS response associated with DNA damage is well known to promote biofilm formation^63^. We show here that ParE^I^ expression induces biofilm formation in *E. coli* (Fig. S6) and characteristic RNA signatures of the SOS response. Biofilms have increased resistance to antimicrobial agents^64,65^ and stress responses that stimulate biofilm formation may be expected to enhance resistance to the host immune system.

In summary, ParDE^I^ appears necessary and sufficient to stabilise important (mostly IncI and IncF-type) conjugative antibiotic resistance plasmids in bacterial cells. Importantly, this type II TAS is also induced by and strongly protects bacteria from insults normally associated with a RecA-dependent SOS response (heat, quinolone and aminoglycoside antibiotics). ParDE^I^ contributes to biofilm formation and allows survival *in vitro* at antibiotic concentrations that exceed those that can be achieved *in vivo* by several orders of magnitude. This high-level antibiotic tolerance is spread among the top-tier human pathogens, *E. coli* and *Salmonella*, on common self-transmissible antibiotic resistance plasmids and can be expected to promote the survival of these bacterial populations and of the resistance plasmids that protect them.

## Materials and Methods

### Bacterial strains and growth conditions

*E. coli* K12 strains DH5α (*F*−, *80lacZ∆M15, recA1, endA1, hsdR17, phoA, supE44, λ-thi-1, gyrA96, relA1*), UB5201 (*F*−, *pro, met, recA56, gyrA*)^66^, J53 (*F*−, *lac*+*, pro, met*)^67^ and BW25113 (*lacI*q, *rrnB*_T14_, *∆lacZ*_WJ16,_ *hsdR514, ∆araBAD*_AH33,_ *∆rhaBAD*_LD78_) strains were grown on LB broth or LB agar at 37 °C except where stated otherwise.

### Preparation of supplemented M9 media

We suspended 10.5 g of M9 Medium Broth Powder (VWR, AMRESCO, Fountain Parkway, OH) (which contains 6 gm of Na_2_HPO_4_.7H_2_O, 3 gm of KH_2_PO_4_, 0.5 gm of NaCl and 1.0 gm of NH_4_Cl) in 1 L of distilled, deionized water and dissolved with gentle stirring and adjusted the pH to 7.4 with sodium hydroxide. The media was then autoclaved for 15 min at 15 psi at 121 °C. Supplemental components and antibiotics were added to the solution after cooled it to 50 °C. For supplementation, we added 2 mL of 1 M MgSO_4_, 0.1 mL of 1 M CaCl_2_, 50.0 µL of 10 mg/mL FeSO_4_.7H_2_O, 0.1 mL of 10 mg/mL thiamine, 20 mL of 20% glucose and 20 mL of 20% casaminoacids. All supplemental solutions were sterilised by passing through 0.22 µ filter (Millipore). Media were then stored at 4 °C.

### Sequence and structural analysis

BLASTn was used to determine the distribution of TA systems in GenBank data. Amino acid identity and comparison of toxin and antitoxin proteins were performed by ClustalW alignment of MEGA7 software^68^. The secondary structures of toxin and antitoxin were predicted using PSIPRED^69^ software, with default settings.

### Construction of plasmids

Construction of plasmid pJIMK82 are described below and all other plasmids used in this study are described in Table 1. Primers used for plasmids construction, PCR and sequencing are described in Table S2. All the constructs were verified by PCR and sanger sequencing.

**Table 1:** Plasmids used in this study.

Plasmids	Characteristics	Source/Reference
pBCSK+	High copy number cloning vector, Cm^R^	Catalog # 212215, Stratagene
pACYC184	Low copy number cloning vector, Cm^R^, Tet^R^	New England Biolabs, Ipswich, MA; GenBank Accession # X06403
pBAD24	Expression vector with arabinose inducible promoter, Amp^R^, pBR322 replicon	^43^
pBAD33-Gm	Expression vector with arabinose inducible promoter, Gen^R^ marker and p15A replicon	^44^, Addgene ID: 65098
pJIE512b	A naturally occurring antibiotic resistance IncI1 plasmid carrying the ParDE^I^ TA system	^47^
pJIMK77	The ParDE^I^ TA nucleotide sequence with its own putative promoter and RBS was cloned into the *Xba*I and *Bam*HI site of pBCSK^+^	This study
pJIMK86	The ParDE^I^ TA nucleotide sequence with its own putative promoter and RBS was cloned into the *Xba*I and *Bam*HI site of pACYC184	This study
pJIMK78	The *parE*^*I*^ toxin coding sequence was amplified from pJIE512b and cloned into *Eco*RI and *Xba*I sites of pBAD24 plasmid under the control of an arabinose inducible promoter	This study
pJIMK92	The C-terminus truncated *parE*^*I*^ toxin gene (201/282-bp) was cloned into *Eco*RI and *Xba*I sites of pBAD24 under an arabinose inducible promoter	This study
pJIMK56	Derivative of pJIE512b which has an intact copy of ParDE^I^ TA but the large antibiotic resistance region (~28.8 kb) and *pndC* toxin of the PndBCA TA system have been deleted.	^48^
pJIMK82	Derivative of pJIE512b where *parE*^*I*^ toxin, antibiotic resistance region and *pndC* toxin were deleted.	This study
pJIMK99	The *parD*^*I*^ toxin coding sequence was amplified from pJIE512b and cloned into *Eco*RI and *Xba*I sites of pBAD33-Gm plasmid under the control of an arabinose inducible promoter	This study

### pJIMK82

Derivative of pJIE512b where *parE*^*I*^ toxin, antibiotic resistance region and *pndC* toxin were deleted. The deletion of *pndC* region was performed as described previously^48^. ParDE^I^ and the antibiotic resistance region are closely located in the plasmid pJIE512b (Fig. S7) and were deleted in a single deletion event by allelic exchange^70^ using *fosA3*-specific primers, Long-F and Long-R (Table S2), targeting 50-bp upstream and downstream of the target region for deletion.

### Plasmid stability assay

Plasmid stability was assessed as previously described^71^, with minor modifications. A single colony of *E. coli* bacteria carrying plasmid was grown in LB broth at 37 °C with shaking at 225 rpm without antibiotic. Bacterial cultures were transferred into fresh LB medium at 1:1,000 dilution twice daily for 4 days. Samples were taken before every transfer, diluted in saline and plated on to LB agar without antibiotic and incubated at 37 °C for 18 h. From each plate, 120 colonies were replica plated onto LB agar plates with and without relevant antibiotic to estimate plasmid retention.

### Growth assays and bacterial cell morphology

Single colonies of *E. coli* J53 carrying recombinant plasmids pJIMK78 (pBAD24 with *parE*^*I*^ toxin) or pJIMK92 (pBAD24 with truncated *parE*^*I*^) or pBAD24 (vector control) were inoculated into 10 mL LB broth in 100 mL conical flasks, with ampicillin 50 µg/mL and J53 with pJIMK78 and pJIMK99 (*parE*^*I*^ toxin in pJIMK78 and *parD*^*I*^ antitoxin in pJIMK99) was inoculated in to the LB broth with ampicillin 50 µg/mL and gentamicin 8 µg/mL and grown overnight (O/N) at 37 °C with shaking at 225 rpm. Cultures were adjusted to optical density (OD_600_) of 1.00 and diluted at 1:1,000 ratio into fresh LB broth and then inoculated (200 µl) into 96 well plate with 0.2% arabinose, ampicillin and gentamycin as needed. An automated multi-mode microplate reader SpectraMax iD5 (Molecular Devices, LLC; CA) was used to assay results. Bacteria were grown in microplates at 37 °C with medium shaking. Absorbance of the culture was measured at a wavelength of 600 nm (OD_600_) every 10 min over 10 h. Experiments were performed in triplicate and mean and standard deviations of nine data points for each sample were calculated and plotted.

To evaluate the effects of ParE^I^ toxin on exponential growing cells, J53(pJIMK78) or J53(pJIMK92) were inoculated from O/N culture into 10 mL LB broth with ampicillin at 1:1,000 ratio and grown for 4 h at 37 °C with shaking and then 0.2% arabinose (for induction of the *ara* promotor) was added to the culture. To evaluate the neutralisation of toxin by cognate antitoxin, J53(pJIMK78 + pJIMK99) was inoculated from O/N culture into 10 mL LB broth at 1:1,000 ratio with ampicillin and gentamycin and grown for 4 h at 37 °C with shaking and then 0.2% arabinose (for induction of the *ara* promotor) was added. The bacterial cultures were sampled at 0, 30, 60 and 90 min after addition of arabinose, OD_600_ was measured and cell viability calculated by serial dilution onto solid growth media. Bacterial cell morphology was observed under phase contrast using an Olympus BX43 light microscope (Olympus corporation, Tokyo, Japan).

### Determination of minimum inhibitory concentration (MIC)

MICs of ciprofloxacin, gentamicin and cefotaxime antibiotics for *E. coli* strain J53 with and without pBAD24 vector control and derived constructs pJIMK78, pJIMK92, pACYC184 vector control and pJIMK86, pBCSK+ vector control and pJIMK77, pJIMK56, pJIMK82 and pJIMK99, as well as J53 carrying both pJIMK78 and pJIMK99 together, were determined by E-test (BioMérieux, France).

### Antibiotic treated bacterial persister assay

Antibiotic tolerance and bacterial persister formation was assayed in supplemented M9 media described earlier in this section and previously^25^. Single colonies of *E. coli* K12 bacteria J53 carrying plasmids pACYC184 (vector only) or pJIMK86 (pACYC184 with ParDE^I^ TA) or pJIMK82 (derivative of pJIE512b without ParDE^I^) or pJIMK56 (derivative of pJIE512b with ParDE^I^) were grown O/N in 10 mL of supplemented M9 media in 100 mL conical flasks at 37 °C with shaking at 225 rpm with appropriate antibiotics. After O/N growth the cultures were adjusted to OD_600_ = 1.0 and sub-cultured at 1:100 ratio in 10 mL supplemented M9 media and grown at 37 °C with shaking at 225 rpm for about 4 to 4.5 h to reach the OD_600_ at 0.5. Supra-MIC concentrations of ciprofloxacin (5 µg/mL), gentamicin (16 µg/mL) or cefotaxime (16 µg/mL) were added as indicated and continued growth for further 5 h. Bacterial cultures were collected before and after 3 h and 5 h of antibiotic exposure and serial dilutions were plated onto LB agar plates and incubated at 37 °C incubator for 18 h. Colonies were then counted and percent survival and log survival rate calculated.

Direct effects of ParE^I^ in antibiotic tolerance were measured as follows. Single colonies of *E. coli* J53 carrying plasmid pBAD24 (expression vector), or pJIMK78 (*parE*^I^ in pBAD24) or pJIMK92 (truncated *parE*^I^ in pBAD24) were grown O/N in 10 mL of supplemented M9 media in 100 mL conical flasks at 37 °C with shaking at 225 rpm with appropriate antibiotics. After O/N growth the cultures were adjusted to OD_600_ = 1.0 and sub-cultured at 1:100 ratio in 10 mL supplemented M9 media (without glucose, because glucose is a repressor of *ara* promoter; casaminoacids in the media as a carbon source in absence of glucose) and grown at 37 °C with shaking at 225 rpm for about 4 to 4.5 h to reach the OD_600_ at 0.5 and then 0.2% arabinose was added to each culture flask to induce toxin expression. Bacteria were then grown for another 3 h and then supra-MIC concentrations of antibiotic were added and continued growth for further 5 h. Bacterial cultures were collected before and after 3 h and 5 h of antibiotic exposure and survival calculated as above.

### Real time reverse transcriptase assay

mRNA from *recA*, *lexA*, *rpoS* and *parDE*^*I*^ was measured by quantitative reverse-transcriptase (qRT) PCR. For *recA*, *lexA* and *rpoS* gene expression, ParE^I^ toxin was expressed or repressed from J53(pJIMK78) by adding 0.2% arabinose or 0.2% glucose respectively and cells were collected at t = 0, 30, 60, and 90 minutes. Bacteria with the empty vector, J53(pBAD24), and ciprofloxacin (0.25 µg/mL)-treated J53 bacteria were used as negative and positive controls respectively and assayed at the same time points. mRNA from *parDE*^*I*^ in exponentially growing bacterial cultures exposed to sub-MIC concentrations of CIP (0.05 µg/mL), or GEN (0.2 µg/mL), or CTX (0.2 µg/mL) and cells were collected at t = 0, 30, 60 and 90 minutes.

RNA extraction, cDNA preparation and qRT-PCR were performed as previously described^72^. Briefly, total RNA was isolated using NucleoSpin RNA kit (Macherey Nagel, Germany). RNA was treated with DNase (TURBO DNA-*free* Kit, Ambion) and freedom from DNA contamination was further confirmed by PCR. cDNA was synthesized by high-capacity cDNA reverse transcriptase kit (Applied Biosystems). One microgram of the initially isolated RNA was used in each reverse transcription reaction. cDNA was diluted 1:10 and 2 μl were used for PCR (Table S2 for primers). Expression of *E. coli rpoB* was used as a reference. The stability of *rpoB* expression was assessed using the variation of threshold cycle (C_T_) values across samples and experimental conditions. Efficiency comparisons of target gene primers were performed against *rpoB* primers. The 2^−ΔΔC^_T_ (Livak) method^73^ was used to determine the relative difference in expression using the geometric mean of *rpoB* ΔC_T_ values for normalisation. For normalisation of expression, we first normalised C_T_ of the target gene to that of *rpoB* for test samples (samples at different test conditions) and calibrator samples (samples just before exposing in test conditions, here, at 0 min time point). The ΔC_T_ of the test samples were then normalised to the ΔC_T_ of the calibrators and the normalised expression ratio calculated (2^−ΔΔCT^). Mean 2^−ΔΔCT^ values and standard deviations for each target gene were derived in three biological replicates, each with three technical replicates, and presented in the graph.

### Other stress tolerance assays

For heat stress experiments, J53 bacteria with plasmid pACYC184 (vector) or pJIMK86 (cloned *parDE*^*I*^) were grown in supplemented M9 media at 37 °C O/N with appropriate antibiotic and then subcultured in fresh supplemented M9 media at 1:100 ratio and incubated for a further 4 hours. Bacteria were then transferred to 42 °C. Bacterial cultures were sampled at different time intervals (0, 30, 60, 90 min) and dilutions were spread onto LB agar plates and grown at 37 °C for a further 18 h. To examine the direct effect of ParE^I^ toxin on heat stress tolerance, overnight cultures of *E. coli* J53(pJIMK78) and the vector-only control J53(pBAD24) were subcultured in fresh supplemented M9 media without glucose at 1:100 ratio and grown for 4 h, then 0.2% arabinose was added to induce ParE^I^ toxin expression from J53(pJIMK78) and grown for another 3 h. Bacterial cultures were then transferred to 42 °C and sampled at different time intervals (0, 30, 60, 90 min) for viable cell count and for qRT PCR analysis as described above.

For nutrient stress, J53(pACYC184), J53(pJIMK86), J53(pBCSK+) and J53(pJIMK77) bacteria were grown overnight in supplemented M9 media (with/out 0.4% glucose and 0.4% casamino acid supplementation, as specified). Overnight cultures were adjusted to an OD_600_ of 1.0 and subcultured at 1:100 ratio in fresh supplemented M9 media and OD_600_ was subsequently measured hourly for 8 h (no glucose supplementation) and cell viability was calculated for 48 h (no glucose and casamino acid supplementation).

### Biofilm assay

*E. coli* J53(pBAD24), J53(pJIMK78) and J53(pJIMK92) were grown in LB medium with ampicillin at 37 °C overnight. Bacterial cultures were adjusted to OD_600_ = 1.0 and subcultured at 1:100 ratio in LB broth with ampicillin. After 3 h growth, 0.2% arabinose was added to induce toxin expression. After 3 h incubation with shaking at 37 °C, 500 µL of cultures were transferred to polystyrene tubes and incubated at 37 °C without shaking. Biofilm formation was measured by staining with crystal violet as described previously^74^ after 24 h and 48 h and cells were also observed under microscope.

## Supplementary information

Suppelemntary Information

## Data Availability

All data generated or analysed during this study are included in this published article and its Supplementary Information File.
