# Supplementary material for: A ParDE-family toxin antitoxin system in major resistance plasmids of Enterobacteriaceae confers antibiotic and heat tolerance
Source: Sci Rep. 2019 Jul 8;9:9872. doi: 10.1038/s41598-019-46318-1 (PMC6614396; doi:10.1038/s41598-019-46318-1)
Supplement: Supplementary file 1 — Suppelemntary Information [file 41598_2019_46318_MOESM1_ESM.pdf]

Supplementary Information

for

A ParDE-family toxin antitoxin system in major resistance plasmids of Enterobacteriaceae confers antibiotic and heat tolerance

Muhammad Kamruzzaman[1*] and Jonathan Iredell[1,2]

[1]Centre for Infectious Diseases and Microbiology, The Westmead Institute for Medical Research, The University of Sydney, Westmead, New South Wales, Australia;

[2] Westmead Hospital, Westmead, New South Wales, Australia.

***Corresponding Author**: Muhammad Kamruzzaman

E-mail: muhammad.kamruzzaman@sydney.edu.au

Supplementary information:

Table S1: Distribution of ParDE^{I} TA system.

Table S2: Primers used in this study.

Figure S1: Organisation and corresponding protein-protein interactions of toxin and antitoxin genes

Figure S2. The alignment and comparison of amino acid sequence and predicted secondary structure of putative toxin.

Figure S3. The alignment and comparison of amino acid sequence and predicted secondary structure of putative antitoxin.

Figure S4. Comparison of putative promoter region of ParDE^{I} TA system to *E. coli* promoter consensus region.

Figure S5: Role of ParDE^{I} TA system in the antibiotic tolerance/persister cell formation in *E. coli*.

Figure S6. Role of ParE^{I} toxin on *E. coli* biofilm formation

Figure S7. A schematic representation of plasmid pJIE512b.

Table S1. Distribution of ParDE[I] TA system

Sl. no.	Description	Accession no.	Query length coverage	Nucleotide sequence identity	Plasmid replicon type[a]	Source[b]	R/S/V[c]
1	*E. coli* plasmid pCAZ590	LT669764.1	100%	100%	IncI	Bird	R
2	*E. coli* strain MRSN346355 plasmid pMRSN346355_120.3	CP018122.1	100%	100%	IncI	Human	R
3	*E. coli* strain MRSN346638 plasmid pMRSN346638_119.3	CP018116.1	100%	100%	IncI	Human	R
4	*E. coli* strain MRSN346595 plasmid pMRSN346595_120.3	CP018110.1	100%	100%	IncI	Human	R
5	*E. coli* strain MRSN352231 plasmid pMR0716_tem1	CP018104.1	100%	100%	IncI	Human	R
6	*Salmonella enterica* strain SJTUF10584 plasmid pS10584	KX058576.1	100%	100%	IncI1	Food	R
7	*Salmonella enterica subsp. enterica* serovar Heidelberg strain SH14-009 plasmid pSH14-009_99	CP016585.1	100%	100%	IncI	H/A/F	S
8	*Salmonella enterica subsp. enterica* serovar Anatum strain GT-38 plasmid PDM04	CP013224.1	100%	100%	IncI	Turkey	R
9	*Salmonella enterica subsp. enterica* serovar Anatum strain GT-01 plasmid PDM02	CP013221.1	100%	100%	IncI	Turkey	R
10	*Salmonella enterica subsp. enterica* serovar Ouakam strain GNT-01 plasmid pDM01	CP012039.1	100%	100%	IncI	Turkey meat	R
11	*Salmonella enterica subsp. enterica* serovar Newport str. CVM 22462 plasmid pCVM22462	CP009566.1	100%	100%	IncA/C	Dog	R
12	*E. coli* strain E17.16 plasmid pE17.16	CP008733.1	100%	100%	IncI1	Human	R
13	*E. coli* plasmid pJIE512b	HG970648.1	100%	100%	IncI1	Human	R
14	*Salmonella enterica subsp. enterica* serovar Derby plasmid pSD107	JX566770.1	100%	100%	IncI	Pork Meat	R
15	*Salmonella enterica subsp. enterica* serovar Heidelberg plasmid pSH1148_107	JN983049.1	100%	100%	IncI	NF	R
16	*Salmonella enterica subsp. enterica* serovar Typhimurium str. ST4/74 plasmid TY474p2	CP002489.1	100%	100%	IncI	Animal	S
17	*E. coli* plasmid pND11_107	HQ114281.1	100%	100%	IncI	Pig	R
18	*E. coli* ACN001 plasmid pACN001-F	KC853439.1	100%	99%	IncI	Chicken	R
19	*E. coli* plasmid pL2-87	KJ484640.1	100%	99%	IncB/O	Lamb	S
20	*E. coli* B7A plasmid pEB3	CP006001.1	100%	99%	IncFII	Human	S
21	*E. coli* strain SF-173 plasmid pSF-173-1	CP012632.1	99%	99%	IncFII	Human	R
22	*E. coli* strain Ecol_224 plasmid pEC224_2	CP018946.1	100%	99%	IncI	Human	R
23	*Salmonella enterica subsp. enterica* serovar Enteritidis strain SE115 plasmid pSE115	KT868530.1	100%	99%	IncI	Human	R
24	*Salmonella enterica subsp. enterica* serovar Kentucky str. CVM29188 plasmid pCVM29188_101	CP001121.1	100%	99%	IncI	Poultry	R
25	*E. coli* strain ET20160881 plasmid pET20160881	MF078004.1	100%	99%	ND	Human	R
26	*E. coli* strain JB10 plasmid pJB10	KX452392.1	100%	99%	IncI	Turkey	R

27	*E. coli* strain S30 plasmid B	CP010233.1	100%	99%	IncI	Soil	R
28	*Salmonella enterica subsp. enterica* serovar Typhimurium str. CDC 2010K-1587 strain USDA-ARS-USMARC-1908 plasmid pSTY2-2010K-1587	CP016865.1	100%	99%	IncI	Human	R
29	*Salmonella enterica subsp. enterica* serovar Heidelberg strain AMR588-04-00320 plasmid pAMR588-04-00320_99	CP016572.1	100%	99%	IncI	H/A/F	R
30	*Salmonella enterica subsp. enterica* serovar Heidelberg strain AMR588-04-00318 plasmid pAMR588-04-00318_99	CP016568.1	100%	99%	IncI	H/A/F	R
31	*Salmonella enterica subsp. enterica* serovar Heidelberg strain 11-004736-1-7 plasmid p11-004736-1-7_99	CP016516.1	100%	99%	IncI	H/A/F	R
32	*Salmonella enterica subsp. enterica* serovar Typhimurium str. CDC 2010K-1587 plasmid pSTY1-2010K-1587	CP014966.1	100%	99%	IncI	Human	R
33	*Salmonella enterica subsp. enterica* serovar Heidelberg strain N13-01290 plasmid pN13-01290_98	CP012936.1	100%	99%	IncI	Turkey meat	R
34	*Salmonella enterica subsp. enterica* serovar Heidelberg strain 12-4374 plasmid p12-4374_96	CP012929.1	100%	99%	IncI	Human	R
35	*Salmonella enterica subsp. enterica* serovar Heidelberg strain SA02DT10168701 plasmid pSA02DT10168701_99	CP012923.1	100%	99%	IncI	Chicken meat	R
36	*E. coli* strain 369 plasmid p369	KT779550.1	100%	99%	IncI	Chicken	R
37	*E. coli* strain M63 plasmid pESCR	KR494248.1	100%	99%	IncI	Pig	R
38	*E. coli* PCN061 plasmid PCN061p5	CP006641.1	100%	99%	IncI	Pig	R
39	*E. coli* plasmid pCMY2 DNA, strain: TVGHEC01	LC019731.1	100%	99%	IncI	Human	R
40	*E. coli* strain AHC4 plasmid pHNAH4-1	KJ125070.2	100%	99%	IncI	Chicken	R
41	*E. coli* strain C0996A plasmid pCTXM123_C0996	KP198616.1	100%	99%	IncI	Chicken	R
42	*E. coli* strain HV295 plasmid pHV295	KM377240.1	100%	99%	IncI	Chicken	R
43	*E. coli* strain HV292 plasmid pHV292	KM377239.1	100%	99%	IncI	Chicken	R
44	*E. coli* strain HV114 plasmid pHV114	KM377238.1	100%	99%	IncI	Chicken	R
45	*E. coli* N40607 plasmid pTC_N40607	CP007651.1	100%	99%	IncI	Cattle	R
46	*E. coli* FAP1 plasmid unnamed 2	CP009580.1	100%	99%	IncI	Pig	R
47	*E. coli* strain ESBL-305 plasmid pESBL-305	CP008737.1	100%	99%	IncI	Human	R
48	*E. coli* plasmid pC49-108	KJ484638.1	100%	99%	IncI	Chicken	R
49	*E. coli* plasmid pC59-112	KJ484637.1	100%	99%	IncI	Chicken	R
50	*E. coli* plasmid pC60-108	KJ484635.1	100%	99%	IncI	Chicken	R
51	*E. coli* plasmid pH2291-112	KJ484629.1	100%	99%	IncI	Human	R
52	*Salmonella enterica subsp. enterica* serovar Typhimurium plasmid pSTM709 DNA	HG428759.1	100%	99%	IncI	Human	R
53	*Salmonella enterica subsp. enterica* serovar Typhimurium plasmid pSTM2	KF290378.1	100%	99%	IncI	Farm	S
54	*Salmonella enterica subsp. enterica* serovar Typhimurium plasmid pSTM7	KF290377.1	100%	99%	IncI	Farm	R
55	*E. coli* strain B3804 plasmid pIFM3804	KF787110.1	100%	99%	IncI	Pig	R

56	*Salmonella enterica subsp. enterica* serovar Typhimurium strain 9134 plasmid p9134dAT	KF705207.1	100%	99%	IncI	Human	S
57	*Salmonella enterica subsp. enterica* serovar Typhimurium strain 9134 plasmid p9134dT	KF705206.1	100%	99%	IncI	Human	R
58	*Salmonella enterica subsp. enterica* serovar Typhimurium strain 9134 plasmid p9134	KF705205.1	100%	99%	IncI	Human	R
59	*Salmonella enterica subsp. enterica* serovar Heidelberg str. SL476 plasmid pSL476_91	CP001118.1	100%	99%	IncI	Turkey	S
60	*Salmonella enterica subsp. enterica* serovar Anatum str. USDA-ARS-USMARC-1783 plasmid pSAN1-2010K-2577	CP014662.1	100%	98%	IncI	Human	R
61	*E. coli* strain FAM22871 plasmid pFAM22871_1	KU355873.1	100%	98%	IncI	Dairy	R
62	*E. coli* strain YD786 plasmid pYD786-2	KU254579.1	100%	98%	IncFII	Human	R
63	*E. coli* strain EC012 plasmid pEC012	KT282968.1	100%	98%	IncI	Chicken	R
64	*E. coli* strain 2009C-3133 plasmid unnamed3	CP013027.1	100%	98%	IncFII	Human	V
65	*E. coli* plasmid pC271, strain C271	LN735561.1	100%	98%	IncI	Human	R
66	*E. coli* plasmid pV408, strain V408	LN735560.1	100%	98%	IncI	Human	R
67	*E. coli* plasmid pM105, strain M105	LN735559.1	100%	98%	IncI	Human	R
68	*E. coli* plasmid pC193, strain C193	LN735558.1	100%	98%	IncI	Human	R
69	*Salmonella enterica subsp. enterica* serovar Heidelberg str. CFSAN002069 plasmid pCFSAN002069_01	CP005389.2	100%	98%	IncI	Chicken	R
70	*Salmonella enterica subsp. enterica* serovar Heidelberg plasmid pSH696_117	JN983047.1	100%	98%	IncF	Human	R
71	*Salmonella enterica subsp. enterica* serovar Heidelberg plasmid pSH163_120	JN983046.1	100%	98%	IncF	Human	R
72	*Salmonella enterica* plasmid pNF1358	DQ017661.1	100%	98%	IncI	Human	R
73	*Shigella sonnei* plasmid P9 DNA	AB021078.1	100%	98%	IncI/ColIB	Human	S
74	*E. coli* strain D2 plasmid B	CP010139.1	100%	98%	IncI	Dog	S
75	*E. coli* O104:H7 strain RM9387 plasmid pO104_H7	KM085449.1	100%	98%	IncI	Human	S/V
76	*E. coli* strain RM9387 plasmid pO104_H7	CP009105.1	100%	98%	IncI	Cow faeces	S/V
77	*E. coli* plasmid pH1519-76	KJ484631.1	100%	98%	IncF	Human	R
78	*E. coli* plasmid pCERC4	KU578032.1	100%	98%	IncFII	Human	R/V
79	*E. coli* strain 14.3-R4 plasmid pCERC9	KY007017.1	100%	98%	IncFII	Human	S/V
80	*E. coli* strain 11.3-R3 plasmid pCERC5	KU664810.1	100%	98%	IncFII	Human	R/V
81	*E. coli* strain G749 plasmid pG749_1	CP014489.1	100%	98%	IncFII	Human	R/V
82	*Salmonella enterica subsp. enterica* serovar Cerro str. CFSAN001588 plasmid pCFSAN001588_002	CP012835.1	100%	98%	IncI	Cow faeces	S/V
83	*E. coli* strain SF-088 plasmid pSF-088-1	CP012636.1	100%	98%	IncFII	Human	R/V
84	*E. coli* strain DB04277 plasmid pDB4277	KP398867.1	100%	98%	IncFIB	Human	R
85	*E. coli* FAP1 plasmid unnamed 1	CP009579.1	100%	98%	ND	Pig	R

86	*Salmonella enterica subsp. enterica* serovar Kentucky plasmid pCS0010A	CP002090.1	100%	98%	IncF	Chicken	S/V
87	*Salmonella enterica subsp. enterica* serovar Kentucky plasmid pSSAP03302A	CP002089.1	100%	98%	IncF	Chicken	S/V
88	*E. coli* str. S88 plasmid pECOS88	CU928146.1	100%	98%	IncFIB	Human	S/V
89	*Salmonella enterica subsp. enterica* serovar Kentucky str. CVM29188 plasmid pCVM29188_146	CP001122.1	100%	98%	IncFII	Poultry	S/V
90	*E. coli* strain O78-789 plasmid pAPEC-O78-ColV	CP010316.1	100%	98%	IncFIB	Poultry	S/V
91	*E. coli* str. ED1a plasmid pECOED	CU928147.1	100%	98%	IncI	NF	S
92	*E. coli* plasmid pCT, strain C159/11	FN868832.1	99%	98%	IncK	H/A	R
93	*E. coli* strain AR_0162 plasmid tig00003056	CP021681.1	100%	97%	IncF	Human	R
94	*E. coli* strain Ecol_545 plasmid pEC545_3	CP018973.1	100%	97%	IncFIC	Human	R
95	*E. coli* strain MDR_56 plasmid unnamed4	CP019910.1	100%	97%	IncFIC	Human	R
96	*E. coli* strain HYEC7 plasmid pHYEC7-110K	KX518744.1	100%	97%	IncFIB	Pig	R
97	*E. coli* strain MRSN346355 plasmid pMRSN346355_67.9	CP018123.1	100%	97%	IncFIB	Human	R
98	*E. coli* strain MRSN346638 plasmid pMRSN346638_67.9	CP018117.1	100%	97%	IncFIB	Human	R
99	*E. coli* strain MRSN346595 plasmid pMRSN346595_67.9	CP018111.1	100%	97%	IncFIB	Human	R
100	*E. coli* strain MRSN352231 plasmid pMR0716_PSE	CP018105.1	100%	97%	IncFIB	Human	R
101	*E. coli* plasmid pMEX01	KU695535.1	100%	97%	IncF	Cattle	S
102	*Salmonella enterica subsp. enterica* serovar Heidelberg strain SA02DT09004001 plasmid pSA02DT09004001_101	CP016522.1	100%	97%	IncI	Chicken Meat	R
103	*Klebsiella pneumoniae* strain KP04 plasmid pKP04CTXM	KU318420.1	100%	97%	IncFII	Human	R
104	*E. coli* strain S51 plasmid pS51_1	CP015996.1	100%	97%	IncI	Poultry meat	R
105	*Citrobacter freundii* strain AC2901 plasmid AC2901	KU987452.1	100%	97%	IncFII	Clinical	R
106	*E. coli* plasmid pEC13	KU932024.1	100%	97%	IncFII	Human	R
107	*E. coli* str. Sanji plasmid pSJ_82	CP011065.1	100%	97%	IncFII	Pheasant	R
108	*E. coli* O25b:ST131 str. JIE186 plasmid pJIE186-2	JX077110.1	100%	97%	IncFIA,FIB	Human	S/V
109	*E. coli* plasmid pHK17a	JF779678.1	100%	97%	IncF	Pig	R
110	*E. coli* strain G1 plasmid pHK08	JN087529.1	100%	97%	IncF	Human	R
111	*E. coli* strain C017e-caz-1 plasmid pHK09	JN087528.1	100%	97%	IncF	Human	R
112	*E. coli* strain Combat2D2 plasmid pHK01	HM355591.2	100%	97%	IncF	Human	R
113	*Klebsiella pneumoniae* plasmid pKF3-70	FJ494913.1	100%	97%	IncF	Human	R
114	*E. coli* strain H30 plasmid pO26-L	FJ449539.1	100%	97%	IncF	Human	S/V
115	*E. coli* plasmid pAPEC-O2-R	AY214164.3	100%	97%	IncF	Chicken	R
116	*E. coli* strain Ecol_AZ159 plasmid pECAZ159_1	CP019007.1	100%	97%	IncFIC	Human	S/V
117	*E. coli* strain M19 plasmid A	CP010222.1	100%	97%	IncFII	Mouse faeces	S/V

118	*E. coli* strain M15 plasmid A	CP010214.1	100%	97%	IncFII	Mouse	S
119	*E. coli* strain M11 plasmid A	CP010207.1	100%	97%	IncFII	Mouse	S
120	*E. coli* strain M9 plasmid A	CP010197.1	100%	97%	IncFII	Mouse	S
121	*E. coli* strain M6 plasmid A	CP010187.1	100%	97%	IncFII	Mouse	S
122	*E. coli* plasmid pEC14I	KU932025.1	100%	97%	IncFII	Human	R
123	*E. coli* O119:H6 plasmid pEC404/03-3 DNA, strain: EC404/03	AP014806.1	100%	97%	IncI	Human	S/V
124	*Salmonella enterica subsp. enterica* serovar Typhimurium strain 33676 plasmid p33673_IncF	CP012683.1	100%	97%	IncFII	Human	R
125	*E. coli* strain EQ011 plasmid pEQ011	KF582523.1	100%	97%	IncF	Equine	R
126	*E. coli* plasmid pEC_B24	GU371926.1	100%	97%	IncFII	Human	R
127	*Shigella sonnei* plasmid pEG356	FN594520.1	100%	97%	IncFII	Human	R
128	*E. coli* plasmid E873p3, strain E873	LT174529.1	100%	97%	IncFII	Human	S/V
129	*E. coli* plasmid pH2332-166	KJ484626.1	100%	97%	IncFIB	Human	R
130	*E. coli* plasmid pVM01	EU330199.1	100%	97%	IncF	Poultry	S/V
131	*E. coli* isolate WI2 isolate, plasmid: pWI2-incFII	LT838203.1	99%	97%	IncFII	Human	S
132	*E. coli* isolate WI1, plasmid: pWI1-incFII	LT838198.1	99%	97%	IncFII	Human	S
133	*E. coli* strain 2016C-3936C1 plasmid unnamed 5	CP018775.2	100%	97%	IncFIB	Human	R
134	*E. coli* APEC O1 plasmid pAPEC-O1-ColBM	DQ381420.1	100%	97%	IncFIIA	Turkey	S/V
135	*E. coli* F18+ strain EC2173 plasmid pTC1	CP000913.1	99%	97%	IncF	Pig	R/V
136	*E. coli* strain MRSN346355 plasmid pMRSN346355_61.1	CP018125.1	99%	97%	IncFII	Human	R
137	*E. coli* strain MRSN346638 plasmid pMRSN346638_61.1	CP018119.1	99%	97%	IncFII	Human	R
138	*E. coli* strain MRSN346595 plasmid pMRSN346595_62.2	CP018113.1	99%	97%	IncFII	Human	R
139	*E. coli* strain MRSN352231 plasmid pMR0716_IncFII	CP018107.1	99%	97%	IncFII	Human	R
140	*E. coli* SE11 plasmid pSE11-2 DNA	AP009242.1	99%	97%	IncFII	Human	S
141	*E. coli* O55:H7 strain 122262 plasmid	KX808482.1	99%	97%	IncFIB	Human	S/V
142	*E. coli* strain 2013C-4465 plasmid unnamed1	CP015242.1	99%	97%	IncF	Human	S/V
143	*E. coli* O55:H7 str. RM12579 plasmid p12579_2	CP003111.1	99%	97%	IncF	Human	S/V
144	*E. coli* O55:H7 str. CB9615 plasmid pO55	CP001847.1	99%	97%	IncFIB	Human	S/V
145	*E. coli* strain AR_0114 plasmid unitig_2	CP021734.1	98%	97%	IncFIB	Human	R
146	*E. coli* strain 06-00048 plasmid pCFSAN004178P_02	CP012498.1	100%	96%	IncFIB	Vegetable	S
147	*E. coli* strain BK28009 plasmid pBK28009	KU295131.1	98%	97%	IncFIB	Human	R
148	*E. coli* strain ESBL-315 plasmid pESBL-315	CP008738.1	100%	98%	IncI1	Human	R
149	*E. coli* strain ESBL-283 plasmid pESBL-283	CP008736.1	100%	98%	IncI1	Human	R
150	*Shigella sp.* MO17 plasmid pMO17_54	HE578057.1	100%	96%	IncX	Human	S
151	*E. coli* plasmid pSCE516-4	KX023259.1	90%	98%	ND	Chicken	R
152	*E. coli* plasmid pAPEC-O2-ColV	AY545598.5	100%	98%	IncF	Bird	S/V

153	*E. coli* ETEC H10407 p948 plasmid	FN649418.1	86%	97%	IncFII	Human	S/V

[a]Inc, plasmid incompatibility type; ND, not determined

[b]H/A/F, Human or Animal or Food; NF, the source information is not found

[c]R, resistance; S, sensitive; and V, virulence properties of the plasmids

Table S2. Primers used in this study

Primer	Sequence (5′-3′)[a,b]	Target/Comments	Source
ParDE-F-XbaI	gatctagaTCGGCGGTTTTACTCCGGTA	to amplify ParDE^{I} TA system with *Xba*I and *Bam*HI sites	HG970648
ParDE-R-BamHI	acggatccACGTCTGGTGGCCAGCTCAT		
ParE-F-EcoRI	ctgaattcAAGGTTGCCGGTAAATGATG	to amplify *parE*^{I} toxin gene with *Eco*RI and *Xba*I sites	HG970648
ParE-R-XbaI	gctctagaCGTCTGGTGGCCAGCTCAT		
ParD-EcoRI-F	gcgaattcTAGTAATGACGAGGTGATAA	to amplify *parD*^{I} anti-toxin gene	HG970648
ParD-XbaI-R	gctctagaTCATTTACCGGCAACCTTCCT		
ParE201-R-XbaI	gctctagaAAATGCGGGTGAATAACCA	to amplify *parE*^{I} toxin gene	HG970648
FosA3-F2	ATAGAGCGGGGATTAGTGTGG	to amplify *fosA3* gene with native promoter	JF411006
FosA3-R1	GCTGTGGATCTGCACGTTGAA		
Long-F	GTGTGGAAGGTACCCGGGAACTGGTTATTCACCCGCATTTTGTTCTGGTT**ATAGAGCGGGGATTAGTGTGG**	to amplify fosA3 gene with 50 bp flanking region from upstream of the *parE* and downstream of the *bla*$_{CMY-2}$ region	HG970648
Long-R	GACGGGCAAAATGCGCATGGGATTTTCCTTGCTGTATTTTTGTTAAGTGT**GCTGTGGATCTGCACGTTGAA**		
TetA-F	GAGCCTCTACGCCGACCTCA	to amplify *tetA* gene of plasmid N3 with native promoter	FR850039
TetA-R	GTAGCCGGAAGTCGCCTTGA		
U-pndA-F	TTACCGGTCATGGCAAGCAG	in pJIE512b *trbA* gene, upstream of *pndA*	HG970648
U-pndA-R	TGAGGTCGGCGTAGAGGCTCTTAACTTCGTAGGCTAACGTTGCCACAA	at the end of *pndC*, overlapping with *pndA*	
D-pndA-F	TCAAGGCGACTTCCGGCTACAGCTCCAGCCGAATGCCTTT	in hypothetical gene, downstream of *pndA*	HG970648
D-pndA-R	CCGATGGATATCACGGCAGA	in non-coding region, downstream of *pndA*	
rpoB-F-RT	GTAAGGCACAGTTCGGTGGT	*E. coli rpoB* for qRT-PCR	Kamruzzaman et al, 2015
rpoB-R-RT	ATTTCCTGCAGGGTGTATGC		
ParDE-F-RT	ATCGACGCTGAACTGAAGGATG	ParDE^{I} TA for qRT-PCR	HG970648
ParDE-R-RT	GTTCAACGGTCTGGCGCATA		
rpoS-F-RT	GGCGTTGCTGGACCTTATCG	*E. coli rpoS* for qRT-PCR	U00096.3
rpoS-R-RT	TCAATCGTCTGGCGAATCCA		
recA-F-RT	TCCGGTAAAACCACGCTGAC	*E. coli recA* for qRT-PCR	U00096.3
recA-R-RT	CGTGCGTAGATTGGGTCCAG		
lexA-F-RT	CGCGGCTGAAGAACATCTGA	*E. coli lexA* for qRT-PCR	U00096.3
lexA-R-RT	GCGGCAACCCTTCTTCCTCT		

[a]Bold indicates nucleotides overlapping with *fosA3* primers; underlined indicates nucleotides overlapping with *tetA* primers

[b]Lowercase indicates additional nucleotides with *Xba*I (TCTAGA), *Bam*HI (GGATCC), and *Eco*RI (GAATTC) restriction sites designed for cloning.

Ref: Kamruzzaman M, Patterson JD, Shoma S et al. Relative strengths of promoters provided by common mobile genetic elements associated with resistance gene expression in Gram-negative bacteria. *Antimicrob Agents Chemother* 2015; **59**: 5088-91.

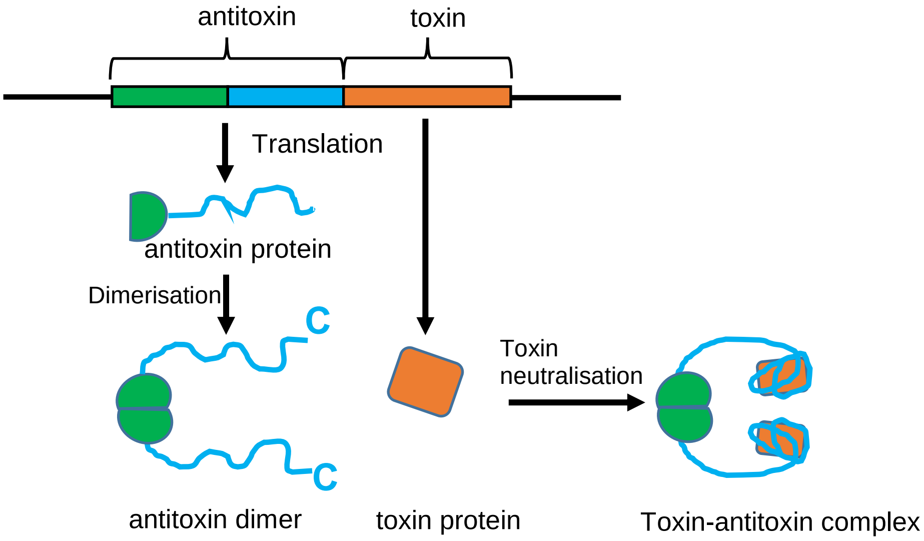

Figure S1. Organisation and corresponding protein-protein interactions of toxin and antitoxin genes commonly found in most type II TA modules, including parDE, relBE, mazEF, ccdAB, where the antitoxin gene is found upstream of the toxin gene. Toxin is coloured orange, N-terminal DNA binding and dimerisation domain of antitoxin is shown in green and C-terminal toxin binding and neutralisation domain is in blue. Adapted with permission from ACS publications (Loris, R. & Garcia-Pino, A. Disorder- and dynamics-based regulatory mechanisms in toxin-antitoxin modules. *Chem. Rev.* **114**, 6933-6947, 2014). Copyright (2014), American Chemical Society.

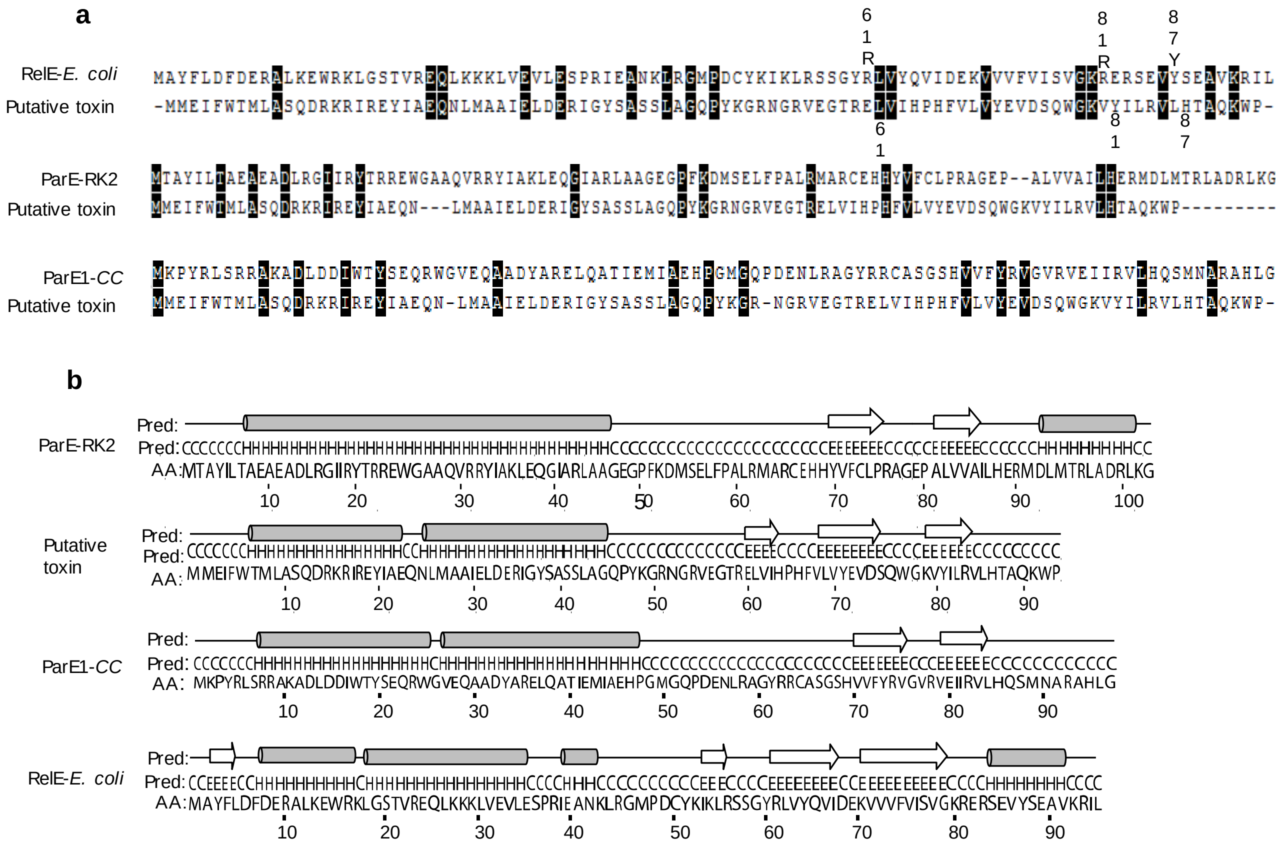

Figure S2. The alignment and comparison of amino acid sequence (**a**) and predicted secondary structure (**b**) of putative toxin with *E. coli* chromosomal RelBE, plasmid RK2 ParDE, and *Caulobacter crescentus* chromosomal ParDE-1 TA system toxins, respectively. Identical amino acids are shown as black shaded. Conserved 61R, 81R and 87Y amino acids in *E. coli* RelE protein and 61, 81 and 87 amino acid positions in the putative toxin are shown. Alignment was performed by ClustalW aligner of MEGA7.0 software. For secondary structure (**b**), arrows represent β-strand, cylinder shapes represent α-helix, and lines represent coil. Pred; predicted secondary structure, and AA; amino acids. Numbers below represent the amino acid positions in the proteins.

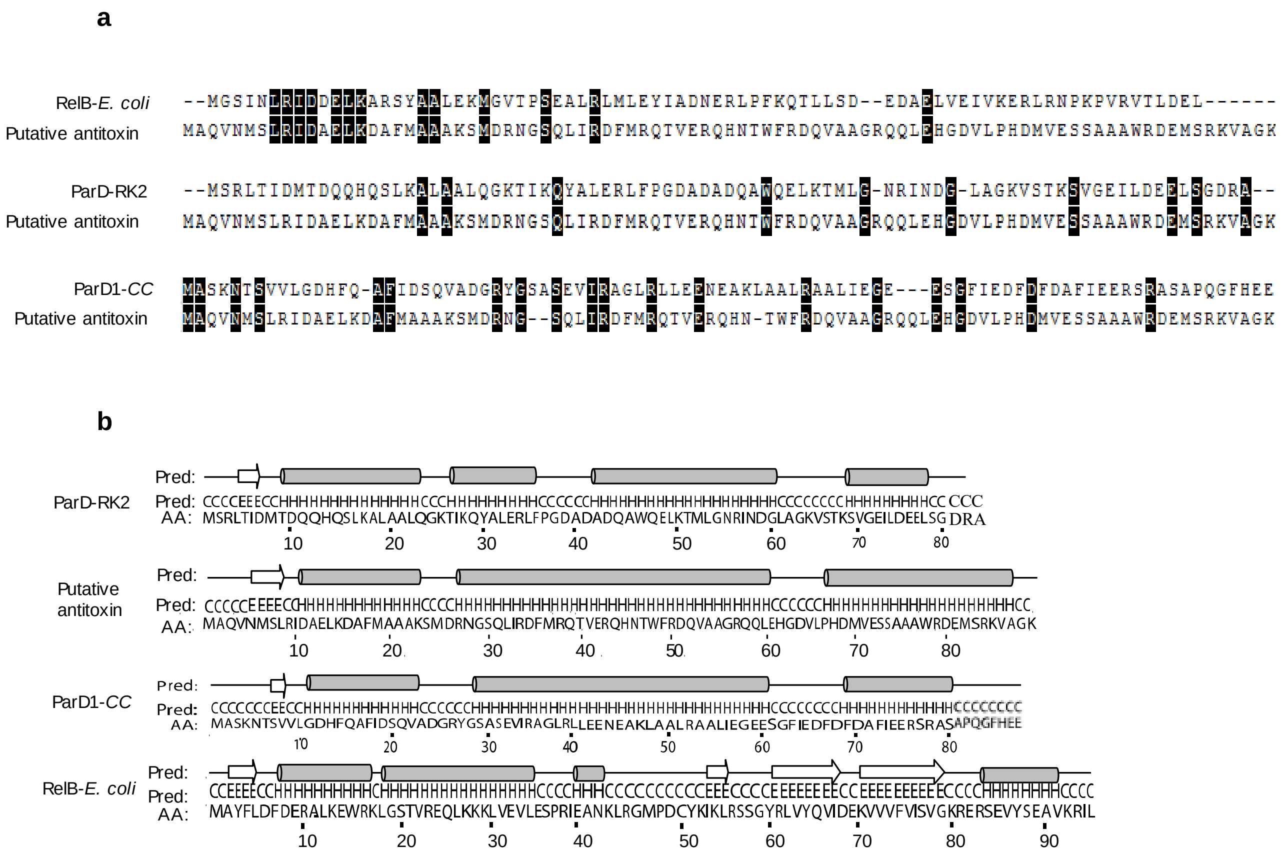

Figure S3. The alignment and comparison of amino acid sequence (**a**) and predicted secondary structure (**b**) of putative antitoxin with *E. coli* chromosomal RelBE, plasmid RK2 ParDE, and *Caulobacter crescentus* chromosomal ParDE-1 TA system antitoxins, respectively. Identical amino acids are shown as black shaded. Arrows represent β-strand, cylinder shapes represent α-helix, and lines represent coil. Pred; predicted secondary structure, and AA; amino acids. Numbers below represent the amino acid positions in the proteins.

-35 -10 RBS

E. coli consensus TTGACA TATAAT AGGAGG

ParDE^{I} ATCCTGGTGACGATATCTGAATCGTTATATACTGTGTATATACGTAGTAATGACGAGGTGATAA**ATG**GCA

Figure S4. Comparison of putative promoter region of ParDE^{I} TA system to *E. coli* promoter consensus region. The -35, -10 and ribosome binding site (RBS) are shown in box, non-identical nucleotides are shaded grey and start codon is in bold.

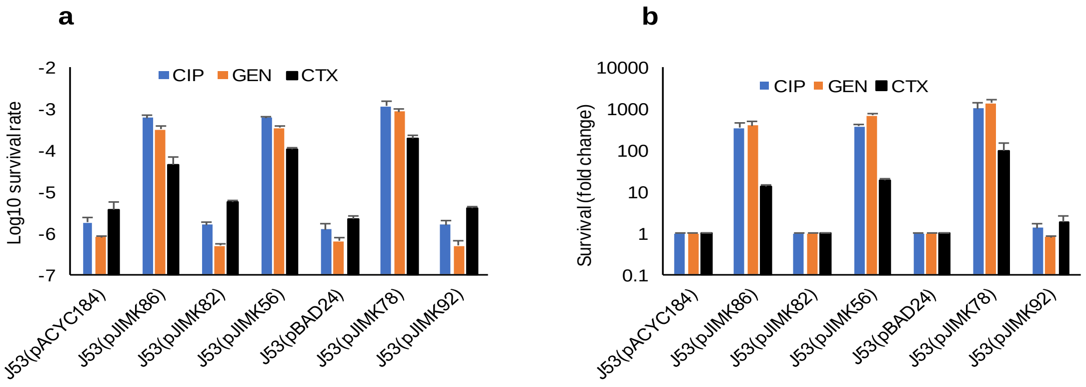

Figure S5. Role of ParDE[I] TA system in the antibiotic tolerance/persister cell formation in *E. coli*. (**a**) Bacterial survival after 3h treatment of high doses of antibiotics; CIP (■), GEN (■) and CTX (■) antibiotics. *E. coli* carrying ParDE[I] [in cloned plasmid J53(pJIMK86) or natural plasmid J53(pJIMK56) or activation of cloned ParE[I] toxin J53(pJIMK78)+ 0.2% arabinose] increased the bacterial survival rate and (**b**) estimated ~ few hundreds to1000-folds against CIP and GEN and ~ 10 to 100-folds against CTX antibiotic. Experiments for tolerance to different antibiotics (**a**, **b**) are repeated five times and mean values and standard deviations (error bars) are represented in the graphs.

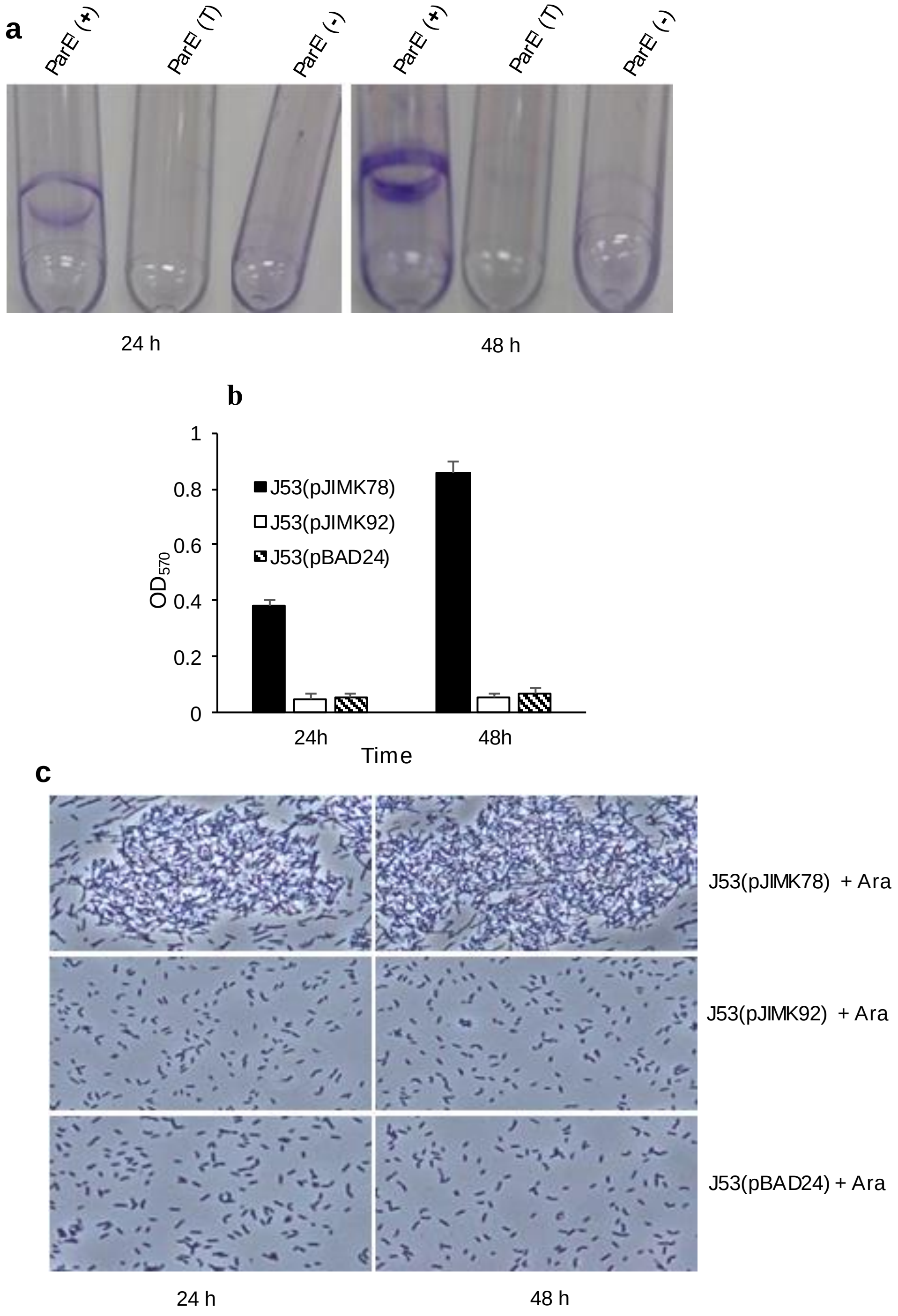

Figure S6. Role of ParE^{I} toxin on *E. coli* biofilm formation. *E. coli* J53(pJIMK78) strain produced biofilm on polystyrene tube surface in the LB medium at 37 ºC under static conditions upon activation of ParE^{I} toxin by adding 0.2% arabinose, measured at 24 h and 48 h (**a**). ParE^{I}(+), ParE^{I}(T) and ParE^{I}(-) represented the J53 strain with cloned ParE^{I}, truncated ParE^{I} and vector (no ParE^{I}), respectively. The OD of dissolved biofilm was also measured and presented (**b**). (**c**) Microscopic observation of J53(pJIMK78) upon ParE^{I} toxin activation also shown biofilm masses (top panel), whereas, only planktonic cells were observed from the *E. coli* carrying truncated toxin in pJIMK92 and pBAD24 vector only.

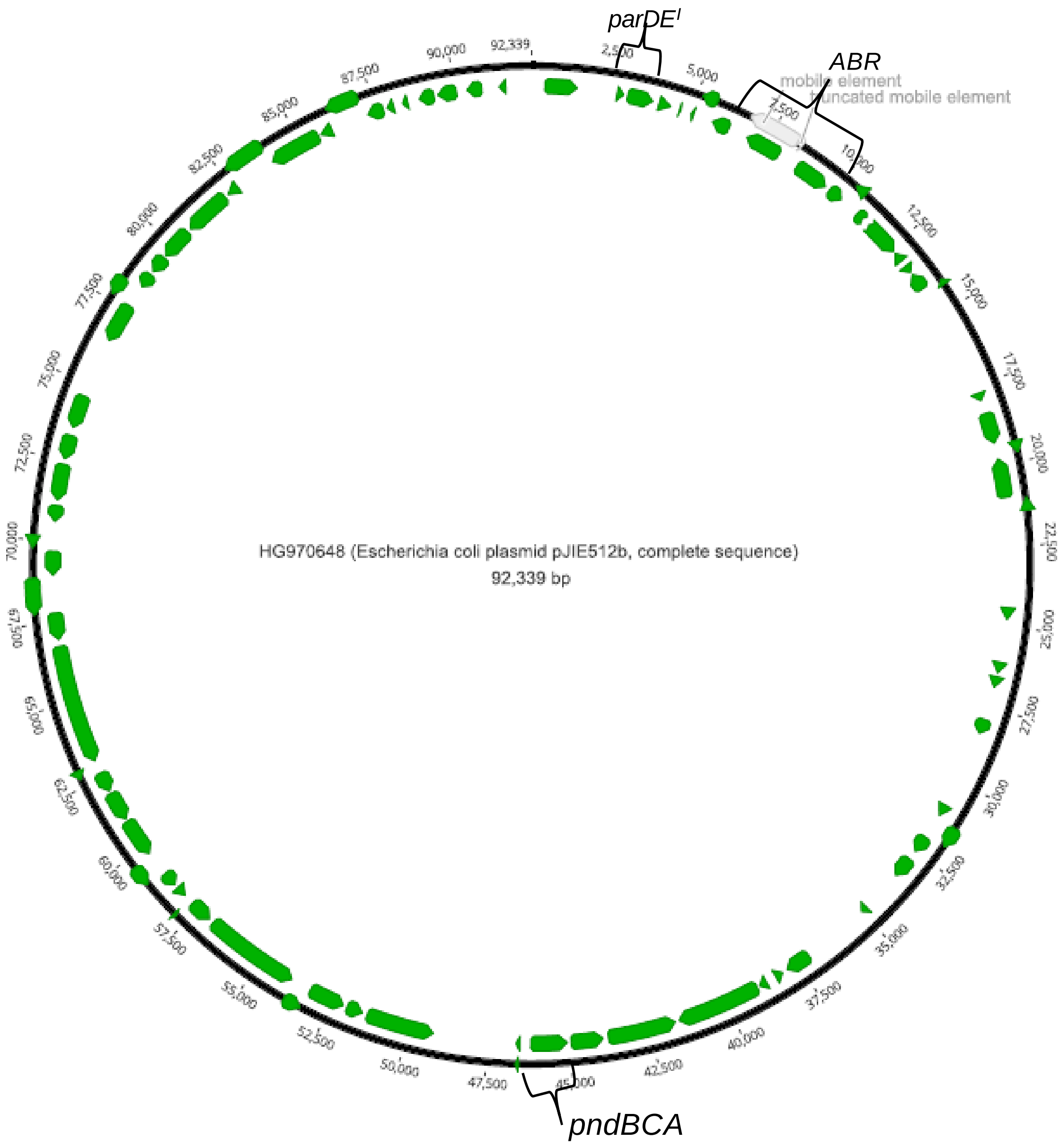

Figure S7. **A schematic representation of plasmid pJIE512b.** The location of the antibiotic resistance region (ABR), *parDE*^{I} and *pndBCA* TA systems on the IncI1 plasmid pJIE512b are shown.
